# Redetermined structure of β-dl-me­thio­nine at 105 K: an example of the importance of freely refining the positions of the amino-group H atoms

**DOI:** 10.1107/S1600536814022223

**Published:** 2014-10-15

**Authors:** Carl Henrik Görbitz

**Affiliations:** a Department of Chemistry, University of Oslo, PO Box 1033 Blindern, N-0315 Oslo, Norway

**Keywords:** hydrogen-bond geometry, amino group, refinement model, β-dl-me­thio­nine, crystal structure

## Abstract

Diffraction data were taken from the contribution named ‘β-dl-Me­thio­nine at 105 K′ by Alagar *et al.* [*Acta Cryst.* (2005 ▶). E**61**, o1165–o1167]. Refinement of the coordinates of the three amino H atoms, previously constrained to an idealized geometry, shows that the amino group is in fact rotated 13.5° from the perfectly staggered orientation. This apparently modest change has a profound impact on the calculated hydrogen-bond geometries.

## Abstract 2

By refining positional coordinates for the three amino H atoms of a previously published amino acid structure, an improved structural model with shorter and more linear hydrogen bonds is obtained.

## Chemical context   

**Figure 1:**
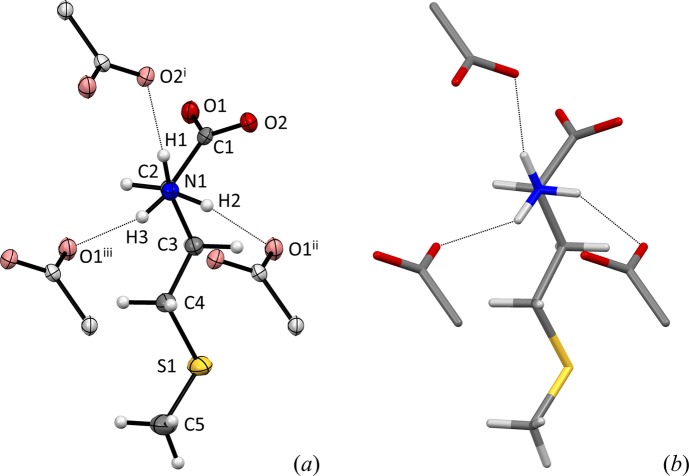
(*a*) The structure of dl-me­thio­nine, (I), viewed approximately along the N1—C2 bond vector, with 50% probability thermal displacement ellipsoids. The racemate contains mol­ecules of both hands; the one depicted here is the d-enanti­omer. Carboxyl­ate groups of three neighboring amino acids accepting hydrogen bonds are shown in a lighter tone. O2^i^ is at (−*x*, *y* + 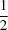, −*z* + 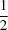), O2^ii^ at (*x* + 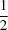, −*y*, *z*) and O^iii^ at (*x* + 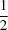, −*y* + 1, *z*), see Table 2 ▶. Compared to the previously published structure shown in capped sticks representation in (*b*) (Alagar *et al.*, 2005 ▶), the amino group has been rotated clockwise by about 13.5° to give shorter and more linear hydrogen bonds.

Upon comparing the hydrogen-bond geometries of the high-temperature α-phase of the amino acid racemate dl-me­thio­nine (Görbitz *et al.*, 2014 ▶) with the best published structure of the β-phase [Alagar *et al.*, 2005 ▶; refcode DLMETA05 in the Cambridge Structural Database (CSD), Version 5.35; Allen, 2002 ▶], we noted that H⋯O distances surprisingly appeared to get shorter at 340 K than at 105 K. This was judged to be an artefact resulting from different ways of handling the amino H atoms. Alagar *et al.* (2005 ▶) used an idealized geometry and a perfectly staggered orientation for this group in their refinement; while we found a 14° counterclockwise rotation (for the l-enanti­omer) that served to give three shorter and more linear inter­actions. The experimental and structural data of Alagar *et al.* (2005 ▶), with coordinates for the d-enanti­omer as the asymmetric unit, were subsequently downloaded and refined again with free amino H atoms, thus increasing the number of parameters from 82 (nine parameters for nine atoms + scale factor) to 91. In the improved structural model displayed in Fig. 1 ▶ [*R*(*F*) = 0.0377 *versus* 0.411 and *wR*(*F* ^2^) = 0.0918 *versus* 0.1001], the amino group is shifted slightly away from the staggered orientation through a 13.5° clockwise rotation (for the d-enanti­omer), Table 1 ▶. 
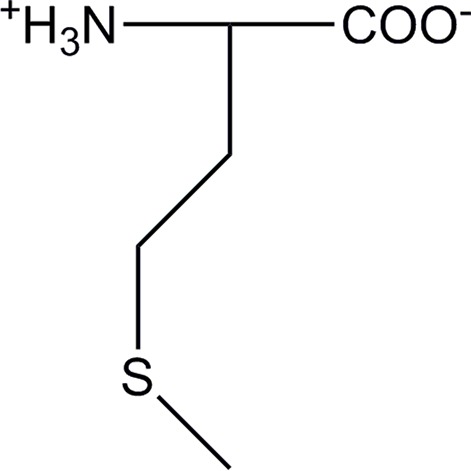


## Supra­molecular features   

The hydrogen-bond geometries listed in Table 2 ▶ show that the free refinement of amino-group H atoms gives close to linear N—H⋯O inter­actions with substanti­ally shorter H⋯O distances. There are no significant changes for geometric parameters involving only C, N and O atoms. This example demonstrates that in order not to unduly bias the statistics of hydrogen-bond geometries in the CSD, it is imperative that H atoms of amino groups and other hydrogen-bond donating functional groups whenever possible are refined in a normal manner and not constrained to theoretical positions. The data set used here (Alagar *et al.*, 2005 ▶) is of good, but not excellent quality. Nevertheless, H atoms can be refined with decent accuracy [standard uncertainties (s.u.’s) = 0.03 Å for N—H distances], allowing experimental determination of hydrogen-bond geometries. In the event that s.u.’s get much higher and/or N—H distances are clearly unreasonably short or long, a rigid rotation refinement of the group (*e.g.* by an AFIX 37 command in *SHELXL*; Sheldrick, 2008 ▶) should be performed. The results of such a refinement for (I), which adds just a single refinement parameter compared to DLMETA05, but reaches the same *R* factor as for (I), are included in Table 2 ▶. The listed values are very close to those of the unconstrained refinement, but are obviously devoid of s.u.’s for geometric parameters involving H atoms.

Under other circumstances restraints on covalent geometry may be employed. Accordingly, we have found that it is often useful to restrain O—H bond distances and H—O—H bond angles (through the 1–3 distances) during refinement of water mol­ecules in crystal hydrates. For a single mol­ecule with atom labels H1W—O1W—H2W, the appropriate *SHELXL* commands would be DFIX 0.85 0.02 O1W H1W O1W H2W and DFIX 1.35 0.03 H1W H2W (the s.u.’s of 0.02 and 0.03 Å being subject to discussion). Similar approaches may be used for groups like –OH and –NH_2_ for which AFIX 37 commands (or equivalent) are not applicable.

## Experimental   

For crystallization details, see Alagar *et al.* (2005 ▶). Crystal data, data collection and structure refinement details are summarized in Table 3 ▶.

Coordinates were refined for amino H atoms; other H atoms were positioned with idealized geometry, with fixed C—H = 0.98 (meth­yl), 0.99 (methyl­ene) or 1.00 Å (methine). *U*_iso_(H) values were set at 1.2*U*_eq_ of the carrier atom or at 1.5*U*_eq_ for methyl and amino groups.

## Supplementary Material

Crystal structure: contains datablock(s) I, global. DOI: 10.1107/S1600536814022223/hb7289sup1.cif

Structure factors: contains datablock(s) I. DOI: 10.1107/S1600536814022223/hb7289Isup2.hkl

Supporting information file. DOI: 10.1107/S1600536814022223/hb7289Isup3.cml

CCDC reference: 1028065

Additional supporting information:  crystallographic information; 3D view; checkCIF report

## Figures and Tables

**Table 1:** Selected torsion angles ()

N1C2C3C4	54.4(2)	C1C2N1H1	46.5(17)
C1C2C3C4	173.53(15)	C1C2N1H2	75.3(15)
C2C3C4S1	179.23(12)	C1C2N1H3	167.4(15)
C3C4S1C5	175.03(14)		

**Table 2:** Hydrogen-bond geometry (, )

*D*H*A*	Parameter	DLMETA05^*a*^	(I)-rigid^*b*^	(I)
N1H1O2^i^	NH	0.89	0.91	0.88(3)
	HO	1.93	1.88	1.91(3)
	NO	2.788(2)	2.787(2)	2.788(2)
	NHO	162	173	174(2)
N1H2O1^ii^	NH	0.89	0.91	0.94(3)
	HO	2.02	1.92	1.89(3)
	NO	2.814(2)	2.815(2)	2.815(2)
	NHO	148	167	169(2)
N1H3O1^iii^	NH	0.89	0.91	0.92(3)
	HO	2.02	1.91	1.91(3)
	NO	2.794(2)	2.795(2)	2.795(2)
	NHO	144	163	161(2)

Symmetry codes: (i) *x*, *y*+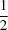, *z*+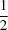; (ii) *x*+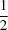, *y*, *z*; (iii) *x*+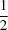, *y*+1, *z*. Notes: (*a*) Alagar *et al.* (2005 ▶), 82 parameters; atoms H1, H2 and H3 were called H1A, H1B and H1C, respectively, by the original authors; the labels used in the CSD entry DLMETA05 have been retained here. (*b*) Rigid rotation refinement of (I), 83 parameters. 0.91 is the standard NH bond length in *SHELXL* (Sheldrick, 2008 ▶) at 105K.

**Table 3:** Experimental details

Crystal data	
Chemical formula	C_5_H_11_NO_2_S
*M*_r_	149.21
Crystal system, space group	Monoclinic, *I*2/*a*
Temperature (K)	105
*a*, *b*, *c* ()	9.877(2), 4.6915(10), 32.603(6)
()	106.25(1)
*V* (^3^)	1450.4(5)
*Z*	8
Radiation type	Mo *K*
(mm^1^)	0.38
Crystal size (mm)	0.32 0.24 0.22

Data collection	
Diffractometer	Bruker SMART CCD area detector
Absorption correction	Multi-scan (*SADABS*; Bruker, 1998 ▶)
*T*_min_, *T*_max_	0.85, 0.92
No. of measured, independent and observed [*I* > 2(*I*)] reflections	6469, 1436, 1373
*R*_int_	0.023
(sin /)_max_ (^1^)	0.623

Refinement	
*R*[*F* ^2^ > 2(*F* ^2^)], *wR*(*F* ^2^), *S*	0.038, 0.092, 1.26
No. of reflections	1436
No. of parameters	91
H-atom treatment	H atoms treated by a mixture of independent and constrained refinement
_max_, _min_ (e ^3^)	0.35, 0.23

Computer programs: *SMART-NT* and *SAINT-NT* (Bruker, 1999 ▶), *SHELXS97*, *SHELXL2013* (Sheldrick, 2008 ▶) and *SHELXTL* (Sheldrick, 2008 ▶).

## supplementary crystallographic information

### Crystal data

**Table:**

C_5_H_11_NO_2_S	*F*(000) = 640
*M**_r_* = 149.21	*D*_x_ = 1.367 Mg m^−^^3^
Monoclinic, *I*2/*a*	Mo *K*α radiation, λ = 0.71073 Å
*a* = 9.877 (2) Å	Cell parameters from 1012 reflections
*b* = 4.6915 (10) Å	θ = 2.6–26.1°
*c* = 32.603 (6) Å	µ = 0.38 mm^−^^1^
β = 106.25 (1)°	*T* = 105 K
*V* = 1450.4 (5) Å^3^	Block, colourless
*Z* = 8	0.32 × 0.24 × 0.22 mm

### Data collection

**Table:**

Bruker SMART CCD area-detector diffractometer	1436 independent reflections
Radiation source: fine-focus sealed tube	1373 reflections with *I* > 2σ(*I*)
Graphite monochromator	*R*_int_ = 0.023
Detector resolution: 8.3 pixels mm^-1^	θ_max_ = 26.3°, θ_min_ = 2.6°
Sets of exposures each taken over 0.5° ω rotation scans	*h* = −12→11
Absorption correction: multi-scan (*SADABS*; Bruker, 1998)	*k* = 0→5
*T*_min_ = 0.85, *T*_max_ = 0.92	*l* = 0→40
6469 measured reflections	

### Refinement

**Table:**

Refinement on *F*^2^	0 restraints
Least-squares matrix: full	Hydrogen site location: inferred from neighbouring sites
*R*[*F*^2^ > 2σ(*F*^2^)] = 0.038	H atoms treated by a mixture of independent and constrained refinement
*wR*(*F*^2^) = 0.092	*w* = 1/[σ^2^(*F*_o_^2^) + (0.0233*P*)^2^ + 2.4782*P*] where *P* = (*F*_o_^2^ + 2*F*_c_^2^)/3
*S* = 1.26	(Δ/σ)_max_ = 0.001
1436 reflections	Δρ_max_ = 0.35 e Å^−^^3^
91 parameters	Δρ_min_ = −0.23 e Å^−^^3^

### Special details

**Table:**

Experimental. Diffraction data and experimental conditions are taken from Alagar *et al.* (2005), CSD refcode DLMETA05.
Geometry. All e.s.d.'s (except the e.s.d. in the dihedral angle between two l.s. planes) are estimated using the full covariance matrix. The cell e.s.d.'s are taken into account individually in the estimation of e.s.d.'s in distances, angles and torsion angles; correlations between e.s.d.'s in cell parameters are only used when they are defined by crystal symmetry. An approximate (isotropic) treatment of cell e.s.d.'s is used for estimating e.s.d.'s involving l.s. planes.
Refinement. Refinement of amino H atom coordinates.

### Fractional atomic coordinates and isotropic or equivalent isotropic displacement parameters (Å^2^)

**Table:**

	*x*	*y*	*z*	*U*_iso_*/*U*_eq_	
S1	0.39652 (6)	0.15681 (12)	0.44292 (2)	0.03291 (18)	
O1	−0.14523 (13)	0.2025 (3)	0.31403 (4)	0.0240 (3)	
O2	−0.01841 (13)	−0.0810 (3)	0.28411 (4)	0.0216 (3)	
N1	0.19296 (17)	0.3000 (3)	0.29733 (5)	0.0193 (3)	
H1	0.142 (3)	0.332 (5)	0.2710 (8)	0.029*	
H2	0.238 (2)	0.122 (6)	0.2999 (7)	0.029*	
H3	0.263 (2)	0.436 (5)	0.3054 (7)	0.029*	
C1	−0.03289 (18)	0.1301 (4)	0.30562 (5)	0.0185 (4)	
C2	0.09967 (18)	0.3087 (4)	0.32589 (5)	0.0183 (4)	
H4	0.0719	0.5103	0.3294	0.022*	
C3	0.17639 (19)	0.1831 (4)	0.36982 (5)	0.0214 (4)	
H5	0.1148	0.2038	0.3889	0.026*	
H6	0.1911	−0.0232	0.3664	0.026*	
C4	0.3196 (2)	0.3214 (4)	0.39143 (6)	0.0246 (4)	
H7	0.3068	0.5281	0.3952	0.030*	
H8	0.3837	0.2973	0.3731	0.030*	
C5	0.5659 (2)	0.3326 (5)	0.45830 (7)	0.0350 (5)	
H9	0.6208	0.2627	0.4864	0.053*	
H10	0.6169	0.2916	0.4371	0.053*	
H11	0.5521	0.5388	0.4597	0.053*	

### Atomic displacement parameters (Å^2^)

**Table:**

	*U*^11^	*U*^22^	*U*^33^	*U*^12^	*U*^13^	*U*^23^
S1	0.0351 (3)	0.0337 (3)	0.0231 (3)	−0.0067 (2)	−0.0031 (2)	0.0074 (2)
O1	0.0225 (7)	0.0168 (6)	0.0333 (7)	0.0012 (5)	0.0089 (5)	−0.0003 (5)
O2	0.0253 (7)	0.0156 (6)	0.0222 (6)	−0.0010 (5)	0.0037 (5)	−0.0028 (5)
N1	0.0207 (7)	0.0169 (8)	0.0190 (7)	−0.0010 (6)	0.0035 (6)	0.0008 (6)
C1	0.0213 (8)	0.0138 (8)	0.0181 (8)	0.0006 (7)	0.0019 (7)	0.0034 (6)
C2	0.0212 (8)	0.0128 (8)	0.0208 (8)	0.0005 (7)	0.0059 (7)	−0.0008 (7)
C3	0.0248 (9)	0.0186 (9)	0.0197 (8)	−0.0008 (7)	0.0043 (7)	−0.0002 (7)
C4	0.0273 (10)	0.0216 (9)	0.0213 (9)	−0.0016 (8)	0.0007 (7)	0.0017 (7)
C5	0.0309 (11)	0.0416 (13)	0.0272 (10)	−0.0024 (9)	−0.0008 (8)	0.0012 (9)

### Geometric parameters (Å, º)

**Table:**

S1—C5	1.806 (2)	C2—H4	1.0000
S1—C4	1.8104 (19)	C3—C4	1.536 (2)
O1—C1	1.262 (2)	C3—H5	0.9900
O2—C1	1.245 (2)	C3—H6	0.9900
N1—C2	1.483 (2)	C4—H7	0.9900
N1—H1	0.88 (3)	C4—H8	0.9900
N1—H2	0.94 (3)	C5—H9	0.9800
N1—H3	0.92 (3)	C5—H10	0.9800
C1—C2	1.539 (2)	C5—H11	0.9800
C2—C3	1.538 (2)		
			
C5—S1—C4	100.27 (10)	C4—C3—H5	108.6
C2—N1—H1	108.9 (15)	C2—C3—H5	108.6
C2—N1—H2	109.2 (14)	C4—C3—H6	108.6
H1—N1—H2	111 (2)	C2—C3—H6	108.6
C2—N1—H3	110.5 (14)	H5—C3—H6	107.6
H1—N1—H3	110 (2)	C3—C4—S1	109.80 (13)
H2—N1—H3	107 (2)	C3—C4—H7	109.7
O2—C1—O1	125.67 (17)	S1—C4—H7	109.7
O2—C1—C2	117.19 (15)	C3—C4—H8	109.7
O1—C1—C2	117.03 (15)	S1—C4—H8	109.7
N1—C2—C3	110.09 (14)	H7—C4—H8	108.2
N1—C2—C1	108.59 (14)	S1—C5—H9	109.5
C3—C2—C1	109.25 (14)	S1—C5—H10	109.5
N1—C2—H4	109.6	H9—C5—H10	109.5
C3—C2—H4	109.6	S1—C5—H11	109.5
C1—C2—H4	109.6	H9—C5—H11	109.5
C4—C3—C2	114.57 (15)	H10—C5—H11	109.5
			
N1—C2—C3—C4	54.4 (2)	O2—C1—C2—C3	−87.60 (18)
C1—C2—C3—C4	173.53 (15)	O1—C1—C2—C3	88.98 (18)
C2—C3—C4—S1	179.23 (12)	C1—C2—N1—H1	46.5 (17)
C3—C4—S1—C5	175.03 (14)	C1—C2—N1—H2	−75.3 (15)
O2—C1—C2—N1	32.5 (2)	C1—C2—N1—H3	167.4 (15)
O1—C1—C2—N1	−150.93 (15)		

### Hydrogen-bond geometry (Å, º)

**Table:**

*D*—H···*A*	*D*—H	H···*A*	*D*···*A*	*D*—H···*A*
N1—H1···O2^i^	0.88 (3)	1.91 (3)	2.788 (2)	175 (3)
N1—H2···O1^ii^	0.94 (3)	1.89 (3)	2.815 (2)	169 (2)
N1—H3···O1^iii^	0.92 (2)	1.91 (2)	2.795 (2)	161 (2)
C2—H4···O2^iv^	1.00	2.43	3.244 (2)	138

Symmetry codes: (i) −*x*, *y*+1/2, −*z*+1/2; (ii) *x*+1/2, −*y*, *z*; (iii) *x*+1/2, −*y*+1, *z*; (iv) *x*, *y*+1, *z*.
